# Solar-driven oil spill recovery using a carbon dots/loofah sponge

**DOI:** 10.1039/d5ra08252h

**Published:** 2026-01-12

**Authors:** Le Thi Hau, Tran Thu Thuy, Nguyen Thi Bich Ngoc, Vanthan Nguyen, Vu Thi Hong Ha, Vu Ngoc Hung, Ho-Suk Choi, Nang Xuan Ho, Do Dieu Linh, Van-Duong Dao

**Affiliations:** a Faculty of Biotechnology, Chemistry and Environmental Engineering, PHENIKAA School of Engineering, PHENIKAA University Hanoi 12116 Vietnam duong.daovan@phenikaa-uni.edu.vn; b Faculty of Mechanical, Electrical, and Computer Engineering, Van Lang School of Technology, Van Lang University Ho Chi Minh City Vietnam; c Department of Chemical Engineering and Applied Chemistry, Chungnam National University Daejoen 99 Korea; d Faculty of Vehicle and Energy Engineering, PHENIKAA School of Engineering, PHENIKAA University Hanoi 12116 Vietnam; e High School for Gifted Students, VNU University of Science Hanoi 10000 Vietnam

## Abstract

Oil spills are considered serious environmental disasters, causing various long-term negative impacts on human and marine life. Therefore, the development of absorbent materials to treat oil spills in a safe and environmentally friendly way is essential. Loofah is a widely available natural product in tropical countries, and due to its low cost and biodegradability, it is used in numerous applications, including food, handicrafts, and cleaning sponges. Owing to its superior porosity, loofah shows potential as a sorbent for oil spill treatment. Herein, we report a hydrophobic cellulose sponge based on a plasma-treated/carbon dots/loofah sponge (P-CLS), which is prepared through a carbon dot (CD) coating process, followed by a nonthermal plasma treatment. The surface temperature of P-CLS can reach up to 55 °C under 1 kW m^−2^ irradiation, resulting in a faster oil transfer rate into the loofah's structure. This sponge demonstrates excellent absorption capacities of 6.6 g g^−1^ and 6.0 g g^−1^ in the dark and under 1 sun irradiation, respectively. Furthermore, P-CLS, with a CD-coated surface and a highly porous structure, shows a remarkable ability to release oil naturally, which reduces the energy required for the recovery process. The sponge achieves a self-releasing efficiency of 41.67%. This work provides a promising approach for practical oil spill remediation.

## Abstract 2

Oil spills are considered serious environmental disasters, causing various long-term negative impacts on human and marine life.

## Graphical abstract

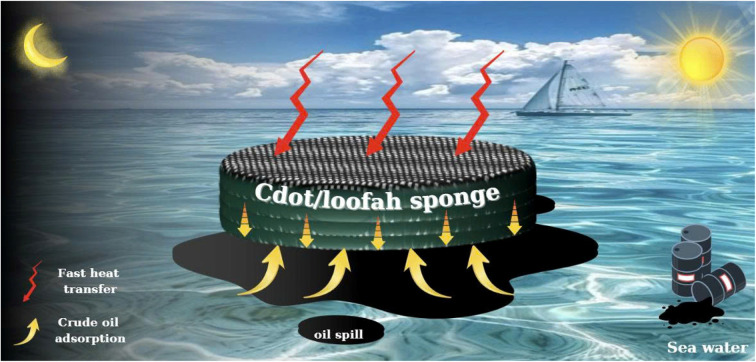

## 1. Introduction

Oil and petroleum products are widely used in various industries. With the rapid growth of global industries, the demand for oil is increasing. Oil transportation entails risks of oil spill accidents, which can cause severe damage to the marine ecosystem and have impacts on multiple sectors, including the economy, human health, society and policies. As reported previously,^1^ as of 2023, approximately 900 kg m^−3^ of oil had accumulated on the ocean surface as a result of oil spill accidents. There are numerous causes of oil spills, including the collision of ships^2^ and the explosion of drilling rigs.^3^ When oil is spilled, it initially spreads out in a thin layer on the seawater surface and then moves with the wind and ocean currents, leaking acute toxins into the aquatic environment.^4^ Depending on weather conditions, the oil may disintegrate, form an oil–water emulsion, evaporate or biodegrade.^5^ In certain situations, hydrocarbons present in the oil are able to generate a toxic sticky emulsion known as “mousse”.^6^ It clings to the bodies of marine animals, impairing respiration, impacting immune function and leading to cardiac dysfunction.^7^ Unfortunately, it cannot be eliminated naturally. In addition, additive substrates in the oil, such as polycyclic aromatic hydrocarbons (PAHs), are major contributors to the mortality of ocean creatures. Owing to their oxidative properties, they attack and bind to DNA and proteins, altering the metabolism of creatures.^6,8^ Moreover, oil pollution poses serious risks to human health through the inhalation of oil vapors or consumption of seafood with accumulated toxins.^7^ It also has extreme impacts on the economy. Specifically, the fisheries sector suffers substantial losses due to the bioaccumulation of toxins in seafood. Meanwhile, coastal tourism and recreational activities may be canceled due to oil slicks, leading to a dramatic decrease in the ocean tourism revenue. Due to the long-term and multifaceted effects of oil pollution, governments and organizations have been striving to develop approaches to manage and remediate its consequences. However, these solutions require a substantial budget over a prolonged period.^7,9^

Among numerous remediation techniques, biomass-driven sorbents have emerged as promising approaches for oil remediation due to their high efficiency, rapid response, low cost, and environmental compatibility.^10–13^ These properties makes them highly suitable for application in vulnerable ecosystems.^14^ The capillary phenomenon is the primary principle of absorption.^15^ Oil molecules interact with the surface of sorbents primarily by van der Waals forces, hydrophobicity, steric interactions and π–π interactions.^4^ Various research studies on the fabrication of biomass-derived sorbents to recover spilled oil have been conducted. In particular, El-din *et al.*^16^ successfully fabricated a sorbent for oil removal from banana peels. The obtained capacity value was 5.31 g g^−1^ sorbent under the best conditions of an average particle size of 0.3625 mm, a temperature of 25 °C, an absorption time of 15 minutes and 3.5% brine. Similarly, Wang *et al.*^17^ fabricated a hydrophobic sponge from loofah that could uptake 6 g of dimethylformamide per gram of the sorbent. This capacity is relatively high compared to synthetic sorbents.

It has been proven that the viscosity of oil drastically decreases with an increase in its temperature, which results in easier oil transfer into the sorbents.^16^ In offshore recovery targets, harnessing solar energy represents an innovative strategy to enhance recovery efficiency. CDs exhibit outstanding potential in the field of solar energy.^18–20^ CDs are one-dimensional materials with a size less than 10 nm and unique optical properties, including strong absorption and bright and colorful fluorescence emissions owing to their nanosize.^21^ In detail, CDs have a broad optical absorption spectrum, which is primarily related to π-plasmon electronic transitions, which can be observed in most nanoscale carbon materials.^22^ Studies on the UV-Vis absorption features of CDs reveal that the peaks at 237 nm, 334 nm, and 460–500 nm correspond to the p–p* energy transition of C=C and n–p* energy transitions of C=O and C=N bonds, respectively.^19^ The thermal conversion mechanism of CDs is dominated by localized electron vibrations. Electrons are excited by sunlight and vibrate in their position in the crystal lattice. The energy of the vibrations is converted into thermal energy and heats the surface of materials.^19^ Plasma is defined as partially ionized gases and widely used to modify wetting surface properties.^23^ The ions generated by plasma equipment chemically interact with hydrophilic functional groups on the surface of cellulose fibers, such as O–H, C=C, –COOH and C–O groups, and transform them into hydrophobic functional groups.^24,25^

Oil recovery from sorbents is primarily achieved using mechanical techniques due to their simplicity and effectiveness. However, biomass-derived sorbents exhibit poor elasticity, and mechanical forces lead to the irreversible collapse of their 3D structure, declining the oil absorption capacity. Loofah sponges have superior porosity, enabling these sorbents to absorb a large amount of oil. Despite this characteristic, loofah sponges cannot effectively retain oil within their structures. Consequently, oil is released from the loofah structures once the sponges are detached from the oil phase. Therefore, loofah sponges have potential for application in oil recovery systems driven by gravity and solar energy. This study introduces a novel biodegradable loofah-based sponge modified by plasma treatment and its ability is resulted from large capillaries. The plasma-treated/CDs/loofah sponge (P-CLS) displays a high compatibility with offshore oil recovery applications, especially in sensitive regions. In this work, a 3D-structured hydrophobic sponge based on the cellulose of loofah, which poses excellent photothermal absorption, has been fabricated *via* CD coating, followed by a nonthermal plasma treatment. This biomass-based sorbent shows an ability to recover oil without external energy. This distinctive characteristic makes loofah sponges superior to other biomass-based sorbents.

## 2. Experiment and methodologies

### 2.1. Fabrication of sorbents

**Fig. 1:**
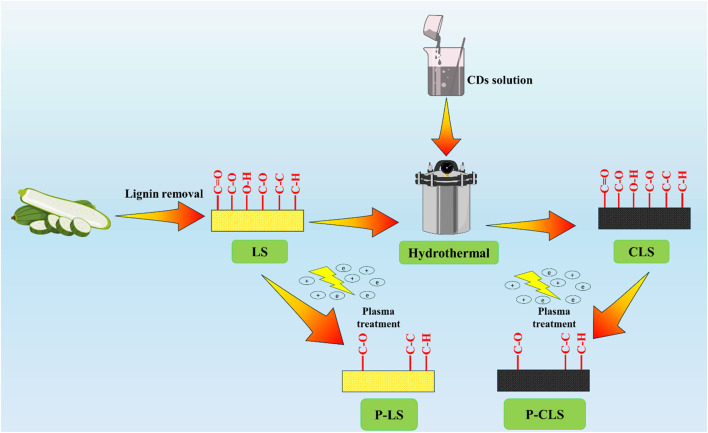
Schematics of the fabrication process of P-CLS and the experimental process.

The loofah sponge (LS) was treated according to the procedure reported in our previous work.^26^ The preparation process is demonstrated in Fig. 1. To completely remove the lignin from loofah cellulose fibers, 500 mL of a mixed solution was prepared by dissolving 60 g of sodium hydroxide (NaOH) and 9 g of sodium sulfide (Na_2_SO_3_). The LS was immersed in the solution and heated at 100 °C for 2 hours. Subsequently, the loofah sponge was washed with deionized water, followed by treating with a solution of 10% H_2_O_2_ at 100 °C for 30 minutes.

The CDs/loofah sponge (CLS) was fabricated *via* the hydrothermal method. 10 g of CDs was dispersed in 125 mL of deionized water. The treated loofah (4 cm × 12 cm) was immersed in the CD solution in a 250 mL Teflon-lined autoclave and treated at 140 °C for 2 h. After the reaction, the obtained sponge was washed thoroughly with distilled water.

To reduce hydrophilic functional groups on the surface of the obtained loofah sponge, a nonthermal plasma process was carried out. The LS and CLS were subjected to plasma treatment using an Ar-driven plasma system under the following experiment conditions: a power of the system of 150 W, a frequency of 150 Hz, an Ar gas flow rate of 5 L min^−1^, an electrode distance of 150 mm, and a treatment duration of 5 minutes. After the process, the hydrophobic plasma-treated/loofah sponge (P-LS) and plasma-treated/CDs/loofah sponge (P-CLS) were obtained.

### 2.2. Material characterizations

The morphologies of loofah sponges were examined by a TESCAN VEGA II LMU scanning electron microscope (SEM). To determine hydrophobic properties, a DataPhysics contact angle (OCA) instrument was used to investigate the contact angle, accompanied by employing Fourier transform infrared spectroscopy (Nicolet Protege 460 FTIR spectrometer) for functional group analysis. Absorption measurements were performed using a UH4150 spectrophotometer (Hitachi).

### 2.3. Oil absorption capability

The absorption capabilities of the loofah sponges were calculated according to the following equation:^27^.
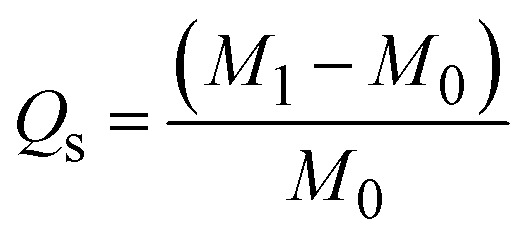
where *Q*_s_ is the absorption capacity (g g^−1^), and *m*_0_ and *m*_1_ indicate the masses of the sponges before and after saturation with oil (g g^−1^), respectively.

The speed (cm s^−1^) of oil uptake into the sponge was assessed using the following equation:
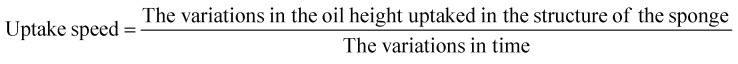


The released oil rates of sponges (g g^−1^) were calculated by the following equation:
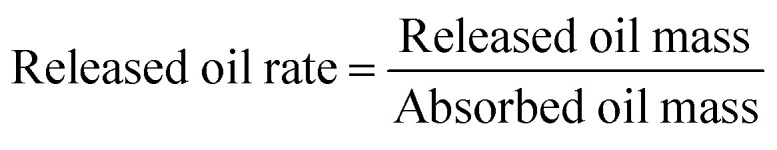


## 3. Results and discussion

### 3.1. Fabrication and characterization of loofah-based sponges

**Fig. 2:**
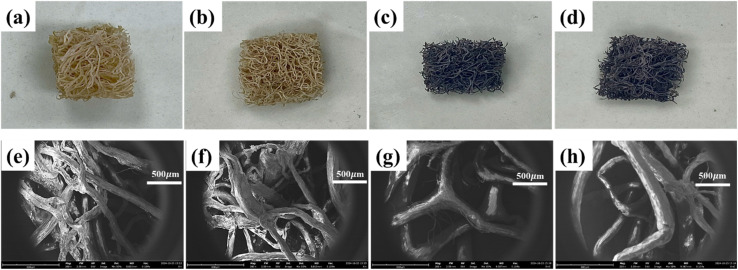
Digital pictures of LS (a), P-LS (b), CLS (c), P-CLS (d) and SEM images of LS (e), P-LS (f), CLS (g), and P-CLS (h).

To examine the surface characteristics of loofah sponges, SEM was employed. Fig. 2 presents the morphologies of the loofah-based sorbent sponges. The raw loofah was chemically treated to remove lignin, followed by CD coating and a plasma process to fabricate hydrophobic sponges for oil spill recovery applications. To carry out experiments, LS, CLS, P-LS and P-CLS were cut into 1 cm × 1 cm pieces. As shown in the digital and optical images, the 3D structure of sponges was formed by the random entanglement of numerous cellulose fibers with large micropores between fibers. The substantial space within the loofah structures endowed the loofah sponges with a low density and a superior oil absorption capacity. No considerable difference in morphologies could be observed in the digital images of LS (Fig. 2a) and P-LS (Fig. 2b) as well as CLS (Fig. 2c) and P-CLS (Fig. 2d). These images revealed that the nonthermal plasma treatment did not alter the cellulose structure of loofah sponges. A visual change was noticeable after the CD coating. The colors of LS and P-LS changed from light yellow to black, implying a broadened absorption band. The morphologies of LS, P-LS, CLS, and P-CLS were further confirmed using scanning electron microscopy (SEM). As can be seen, many micro-sized pores were present on the cellulose fiber surfaces of LS (Fig. 2e), P-LS (Fig. 2f), CLS (Fig. 2g), and P-CLS (Fig. 2h). They played a crucial role in transporting oil within sponges. After the CD coating process, microchannels and micro-sized pores remained intact. These results proved that the CD coating did not change the size of cellulose fibers and pores, thereby preventing any decline in the oil absorption capacity. Moreover, SEM images showed that CDs were uniformly coated onto the fiber surfaces without clogging their microchannels and micro-sized pores. CDs displayed broad-spectrum solar absorption and superior photothermal conversion performance, facilitating the reduction in the viscosity of oil during the recovery process. Meanwhile, the high-porosity structure with numerous microchannels suggested a significant oil absorption capacity and good oil transfer throughout the loofah structures. Furthermore, no remarkable differences in the morphologies of the LS and CLS were observed after plasma treatment. This result further confirmed that the impact of the plasma process on morphologies could be ignored.

**Fig. 3:**
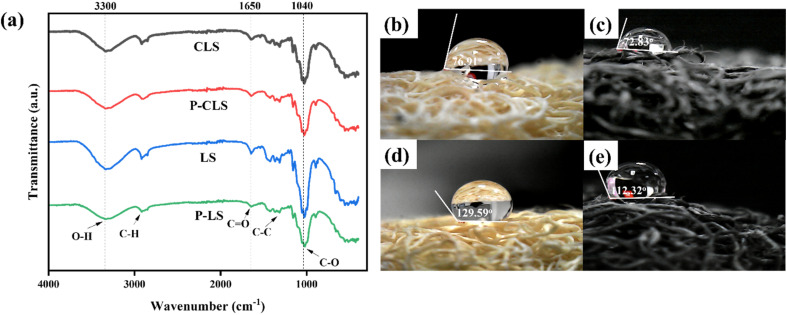
FT-IR spectra (a) and WCAs of LS (b), CLS (c), P-LS (d), and P-CLS (e).

FT-IR spectroscopy was employed to further evaluate the hydrophobic characteristics of LS, P-LS, CLS and P-CLS. In our previous study, the strong interaction between CDs and the cellulose surface was contributed to by the chemical bonding between hydrophilic groups on the surface of CDs and cellulose.^26^ As shown in Fig. 3a, the relative intensity of the absorption peak corresponding to the hydrophilic functional groups of P-LS and P-CLS decreased compared to LS and CLS, respectively. In detail, the absorption intensity of O–H/N–H stretching vibrations at 3100–3300 cm^−1^ decreased significantly after plasma treatment because the plasma beam broke the bonds or partially removed the hydroxyl groups on the cellulose surface. Moreover, the relative intensity of the peak associated with C=O stretching vibrations at 1710 cm^−1^ decreased in P-LS and P-CLS compared to LS and CLS, respectively. The absorption intensity of the C–O stretching vibrations of the pyranose ring of the cellulose structure at 1040 cm^−1^ decreased, indicating that the C–O bonds in the pyranose rings were broken or the pyranose rings rearranged their structures because the plasma process removed some hydroxyl groups of cellulose. There was no new absorption peak had been observed, indicating that the plasma treatment only eliminated functional groups and did not form new groups on the surface of loofah sponges. This evidence confirmed the effectiveness of the nonthermal plasma process in enhancing the hydrophobic properties of cellulose fibers. By reducing the presence of hydrophilic functional groups on the surface of cellulose fibers, the plasma treatment significantly decreased the affinity of the cellulose surface for water molecules, thereby improving the oil–water separation efficiency.

A water contact angle (WCA) system was used to test the hydrophobicity of sponges. As can be observed, water droplets partially absorbed into LS (Fig. 3b) and CLS (Fig. 3c) but stayed on the P-LS (Fig. 3d) and P-CLS (Fig. 3e) and took on a spherical shape. With the elimination of hydrophilic functional groups on the cellulosic surface, the WCA of LS increased from 76.91° (Fig. 3b) to 129.59° (Fig. 3d) after the plasma treatment. Meanwhile, the WCA value of CLS was 72.83° (Fig. 3c), and that of the P-CLS reached 112.32° (Fig. 3e). This indicated that the plasma treatment process enhanced the hydrophobic characteristics of LS and CLS by modifying the surface of sponges. Furthermore, owing to the inherently hydrophilic characteristic of CDs, they contributed to the decrease in the WCAs of the CLS and P-CLS, leading to the lower WCAs of CLS and P-CLS compared to LS and P-LS, respectively. The WCA values of LS, P-LS, CLS, and P-CLS were consistent with the FT-IR analysis, indicating that the enhanced hydrophobicity of P-LS and P-CLS could be attributed to the nonthermal plasma treatment.

**Fig. 4:**
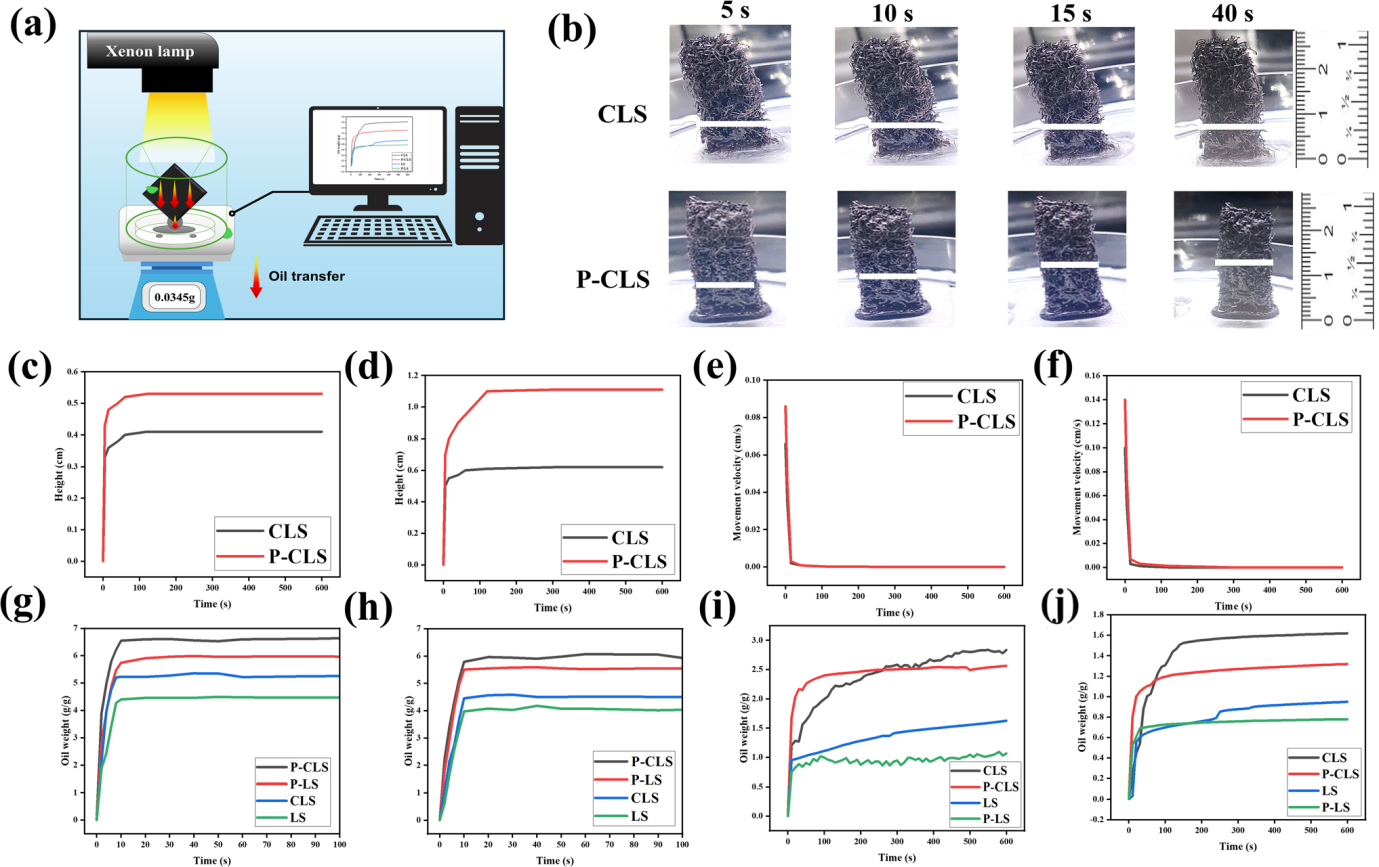
Schematic of the oil-releasing experiment (a). Investigation of the vertical oil absorption rate in the structures of CLS and P-CLS: oil uptake of CLS and P-CLS over time (b); oil height in the structures of CLS and P-CLS in the dark (c) and under illumination (d); oil absorption rates in the dark (e) and under illumination (f); sorption capacities of LS, P-LS, CLS, and P-CLS in the dark (g) and under 1 sun irradiation (h); and the oil weight self-released in the dark (i) and under 1 sun irradiation (j) under gravity.

To recover oil from sorbents, mechanical methods such as squeezing or pressing are commonly employed due to their simplicity and effectiveness. However, these techniques result in irreversible damage to biomass sorbents, leading to a decrease in the oil absorption capacity of sorbents over time. Loofah sponges with a highly porous structure can absorb a large amount of oil. Simultaneously, large pore sizes lead to the ineffective retention of oil within loofah structures. Consequently, the sponges released oil once they were detached from the oil phase. This unique property of loofah sponges allows for energy-free oil recovery while preserving their 3D structure. Fig. 4a demonstrates an oil-releasing experiment, which was conducted under both 1 sun illumination and dark conditions. Loofah sponges were suspended by a thread and placed under a solar simulator. The mass of oil released from the loofah sponges was recorded using an analytical balance.

### 3.2. Oil absorption and self-releasing performance

To evaluate the oil absorption ability, the oil absorption rates of CLS and P-CLS were investigated (Fig. 4b) using vacuum oil VPO-46H. A sponge (1 cm × 2.5 cm × 0.4 cm) was dipped in the oil phase oil. According to the observation, oil was absorbed rapidly upon contact, with the absorption rate being the fastest within the first 5 seconds and reaching saturation after 40 seconds. The P-CLS demonstrated a higher absorption rate and capacity than the CLS sample, as observed in Fig. 4b. In the dark, the oil penetration height for the P-CLS reached 0.53 cm, while for the CLS, it reached 0.41 cm (Fig. 4c), corresponding to maximum absorption rates of 0.086 cm s^−1^ and 0.066 cm s^−1^ (Fig. 4e), respectively. Under a 1 kW m^−2^ light condition, the oil penetration height increased to 1.10 cm and 0.60 cm (Fig. 4d), with maximum absorption rates of 0.14 cm s^−1^ and 0.10 cm s^−1^ (Fig. 4f), respectively. It was observed that the oil penetration height under illumination was lower than that under dark conditions. This phenomenon can be attributed to the photothermal effect of the carbon quantum dot (CD) coating. Upon light exposure, the CDs absorb light and converted it into heat, raising the surface temperature of the material. The increased temperature reduces the viscosity of the oil, facilitating its movement within the porous structure.

However, the elevated temperature simultaneously enhanced the desorption of oil, especially in porous and large-pore structures like loofah. As a result, while oil entered the loofah sponges more easily under illumination, it also escaped more readily, leading to a lower observed oil column height in the CLS and P-CLS compared to dark conditions. These findings highlighted the complex interplay between absorption and desorption influenced by the temperature and confirmed the functional role of the CD coating in modulating the oil absorption behavior of samples under light exposure.

The oil capacities of the LS, P-LS, CLS, and P-CLS (1 cm × 1 cm × 0.4 cm) were evaluated by employing vacuum oil VPO-46H. The material's oil uptake was determined by recording the sample weight at predetermined time intervals during immersion in oil. In the dark, the P-LS and P-CLS sponges could absorb 6.0 and 6.6 g of oil per gram of their weight, respectively. In contrast, LS and CLS could absorb 4.4 and 5.2 g of oil per gram of their weight, respectively (Fig. 4g). Due to their low surface temperature, the viscosity of oil on the surfaces of LS and P-LS did not decrease very much. Consequently, oil molecules faced resistance during absorption into the micropores of cellulose fibers, leading to lower capacities compared to CLS and P-CLS. P-CLS and CLS exhibited 11–18% higher oil capacities than P-LS and LS under the dark condition. When illuminated, the viscosity of the oil drastically decreases. This is beneficial for the absorption of high-viscosity oils by sorbents. However, it also promotes the desorption of some oils from the sorbent's structure. Hence, the capacities of loofah sponges drastically decreased under the 1 sun illumination condition. As can be seen in Fig. 4h, the oil capacities of the LS, P-LS, CLS, and P-CLS were 3.9, 5.5, 4.4 and 6.0 g of oil, respectively. In general, P-LS and P-CLS could absorb more oil than LS and CLS under both dark and irradiation conditions, respectively.

To evaluate the oil release efficiency of the sponges, experiments were conducted according to the simulation model designed, as shown in Fig. 4a. Under 1 sun illumination, the maximum oil-releasing values of LS, P-LS, CLS, and P-CLS samples reached 1.6 g g^−1^, 1.0 g g^−1^, 2.7 g g^−1^, and 2.5 g g^−1^, respectively. Meanwhile (Fig. 4i), the values were 0.9 g g^−1^, 0.7 g g^−1^, 1.6 g g^−1^, and 1.3 g g^−1^ in the dark, respectively (Fig. 4j). The loofah sponges could release oil even in the dark, and their release efficiency increased significantly under illumination. This also showed that the CD coating played a key role in increasing the surface temperature, resulting in the higher oil-releasing rates. Due to their hydrophobic surfaces, P-LS and P-CLS more strongly interacted with oil than PS and CLS, leading to lower oil-releasing rates. The oil-releasing rate was the highest in the first 40 s and then gradually decreased in the next 60 s. The released mass of oil changed slightly after 100 s. These results confirmed the feasibility of applying the material in automated oil recovery systems utilized solar energy. Although the oil recovery rates are lower than those of mechanical methods, this method can save energy during recovery processes and preserve the loofah structure.

**Fig. 5:**
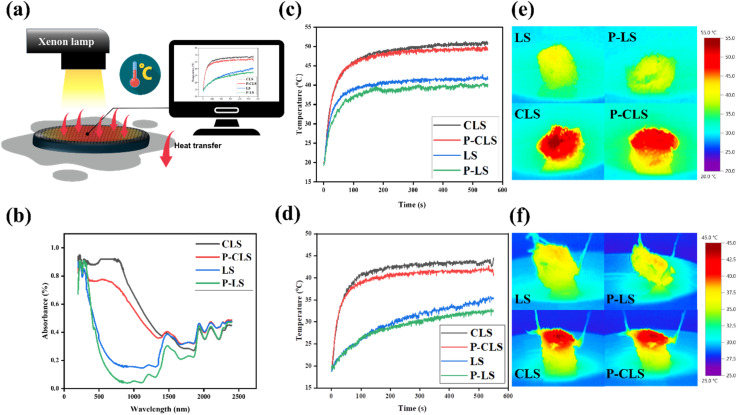
Illustration of temperature investigation (a); UV-vis spectra (b); temperature evoluation in the dry state (no oil) and (c) wet state (d) (in the presence of oil); and IR images of loofah sponges in the dry state (e) and wet state (f).

To investigate the photothermal properties, the surface temperatures of the LS, P-LS, CLS and P-CLS were tested using a thermocouple with a solar simulator at a power density of 1 sun (Fig. 5a). A polystyrene (PS) foam was used to prevent light transmittance and to simultaneously increase the scatterring of light, which enhanced the solar energy harvesting ability of the sorbents.

UV-vis spectroscopy was conducted to investigate the solar absorption capacity and photothermal properties (Fig. 5b). Loofah sponges showed good absorption in the board range of 200–2400 nm. Notably, CLS and P-CLS displayed an excellent light absorption of 80% in the range of 200–800 nm (covering the near ultraviolet and visible regions), while the average absorption of LS and P-LS was about 20% in this light band. A slight decline in light absorption could be observed in plasma-treated sponges (P-LS and P-CLS) compared to untreated sponges (LS and CLS), resulting from the removal of hydrophilic functional groups. However, the value could be negligible.

Fig. 5c illustrates the temperature evolution of the LS, P-LS, CLS and P-CLS under 1 irradiation. The experiment was set up as shown in Fig. 5a. The real-time evolution temperature data were recorded and monitored using a computer. In the absence of oil (dry state), the surface temperatures of the LS, P-LS, CLS and P-CLS were 38.4 °C, 36.5 °C, 47.0 °C and 46.7 °C after 100 s, respectively, and reached saturated temperatures of 41.0 °C, 39.2 °C, 52.0 °C and 48.8 °C after 150 s, respectively. These results confirmed the important role of CDs in harvesting solar energy. Meanwhile, in the presence of oil (wet state) (Fig. 5d), the surface temperatures of the LS, P-LS, CLs and P-CLS reached the values of 24.3 °C, 23.9 °C, 44.0 °C and 42.3 °C after 100 s. Due to the low specific heat capacity of oil, the temperature differences between the dry state and wet state of CLS and P-CLS were relatively small, with a temperature gap of about 8 °C. The temperatures of plasma-treated samples were slightly lower than those of the untreated samples, which is in agreement with the UV-vis and FT-IR results.

Fig. 5e and f show the IR images of the LS, P-LS, CLS, and P-CLS after they reached their maximum temperature. Because the upper surface was in direct contact with the light source, it attained the highest temperature, and the temperature of the samples gradually decreased with the height of the sample; this behavior was consistent with the established heat transfer mechanism. The surface temperature gradient was very uniform, showing that there was uniformity in the structure, and the CD-coated surface experienced uniform heating. The gradient further confirmed the photothermal role of the CD coating, as the temperatures of the CLS and P-CLS in dry and wet states were much higher than those of the other two samples. The maximum temperatures of the plasma-treated samples in both dry and wet states were lower than those of the unplasma-treated samples. This difference may be due to the removal of hydrophilic functional groups through the nonthermal plasma process.

## 4. Conclusion

In this study, we have developed a 3D porous hydrophobic P-CLS for environmentally compatible oil remediation and self-releasing oil recovery. The dual-functional modified carbon dot (CD) coating facilitates broadened light absorption and photothermal conversion, and the nonthermal plasma treatment decreases hydrophilicity and enhances surface oleophilicity, significantly improving interfacial and optical properties. The cellulose porous network provides capillary pathways and structural support, while the CD layer absorbs about 80% of visible light and generates localized heating that influences oil mobility within the capillaries. As a result, the sponge exhibits strong oil uptake under dark and illumination conditions.

Regarding the mechanism, oil penetrates mainly through capillary-driven infiltration, supported by the large pores of cellulose fibers and the oleophilic modified surface. In the dark, P-CLS can absorb about 6.6 g oil per gram of weight, while this value reaches 5.2 g under 1 sun illumination. The self-release behavior arises from gravity-assisted drainage through macropores and photothermal-induced reduction in the oil's viscosity. Further, the P-CLS owns a distinguishing self-releasing oil ability, achieving 41.67% oil removal. This unique feature makes P-CLS outstanding compared to other biomass sorbents. Taking advantage of solar energy, a sustainable renewable energy resource, the oil remediation performance can be enhanced. With its low manufacturing cost, simplicity in fabrication process and high absorption and recovery efficiency with all day accessible work, this study presents a desirable strategy for oil remediation.

## Author contributions

Le Thi Hau: methodology, investigation, data curation, formal analysis, writing – original draft; Tran Thu Thuy: methodology, investigation, data curation, formal analysis, writing – original draft; Nguyen Thi Bich Ngoc: methodology, investigation, data curation, formal analysis, writing – original draft; Vanthan Nguyen: data curation, formal analysis; Vu Thi Hong Ha: data curation, formal analysis; Vu Ngoc Hung: data curation, formal analysis; Ho-Suk Choi: data curation, formal analysis; Nang Xuan Ho: data curation, formal analysis; Do Dieu Linh: data curation, formal analysis; Van-Duong Dao: conceptualization, supervision, project administration, writing – review & editing, funding acquisition.

## Conflicts of interest

The authors declare that they have no financial interests or personal relationships that could have appeared to influence the work reported in this paper.

## Supplementary Material

RA-016-D5RA08252H-s001

## Data Availability

Data available from the authors upon request. Supplementary information (SI) is available. See DOI: https://doi.org/10.1039/d5ra08252h.
