# Supplementary material for: Solar-driven oil spill recovery using a carbon dots/loofah sponge
Source: RSC Adv. 2026 Jan 12;16(3):2221–8. doi: 10.1039/d5ra08252h (PMC12795590; doi:10.1039/d5ra08252h)
Supplement: RA-016-D5RA08252H-s001 [file RA-016-D5RA08252H-s001.pdf]

Supplementary Information (SI) for RSC Advances.
This journal is © The Royal Society of Chemistry 2025

Supplementary material

Solar-driven oil spills recover using Carbon dots/loofah sponge

Le Thi Hau[1], Tran Thu Thuy[1], Nguyen Thi Bich Ngoc[1], Vanthan Nguyen[2], Vu Thi Hong Ha[1], Vu Ngoc Hung[1], Ho-Suk Choi[3], Nang Xuan Ho[4], Do Dieu Linh[5], Van-Duong Dao[1]*

[1]Faculty of Biotechnology, Chemistry and Environmental Engineering, PHENIKAA School of Engineering, PHENIKAA University, Hanoi 12116, Vietnam

[2]Faculty of Mechanical - Electrical and Computer Engineering, School of Technology, Van Lang University, Ho Chi Minh City, Vietnam

[3]Department of Chemical Engineering and Applied Chemistry, Chungnam National University, Daejoen 99, Korea

[4]Faculty of Vehicle and Energy Engineering, PHENIKAA School of Engineering, PHENIKAA University, Hanoi Hanoi 12116, Vietnam

[5]High School for Gifted Students, VNU University of Science, Hanoi 10000, Vietnam

Le Thi Hau, Tran Thu Thuy and Nguyen Thi Bich Ngoc contributed equally to this work

*Corresponding author: Email: duong.daovan@phenikaa-uni.edu.vn

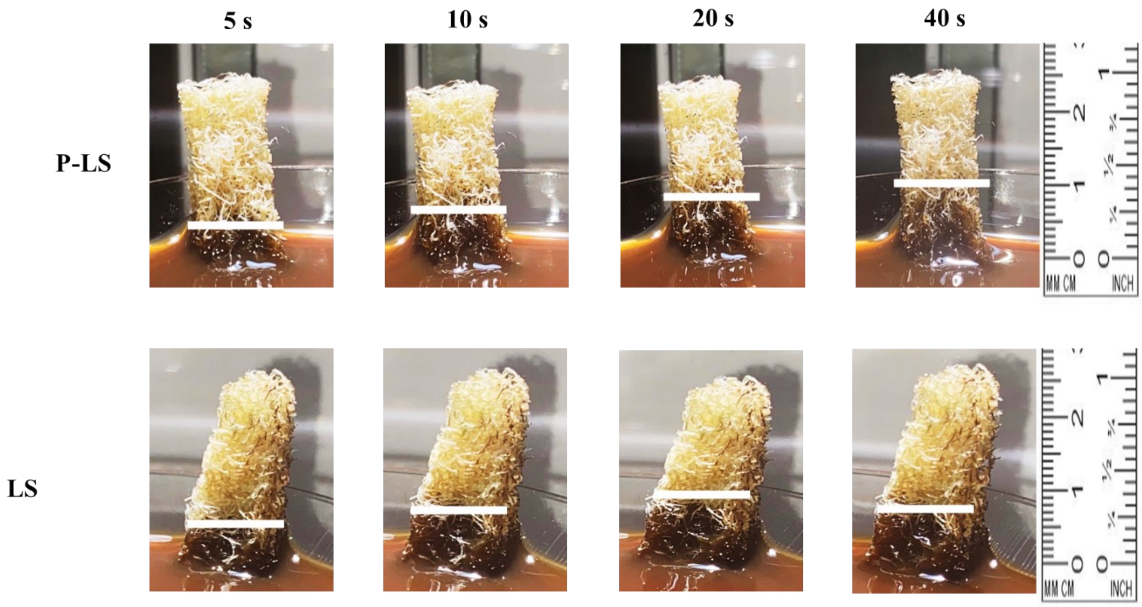

Fig S1. Oil absorption rate of LS and P-LS toward vehicle wasted oil.

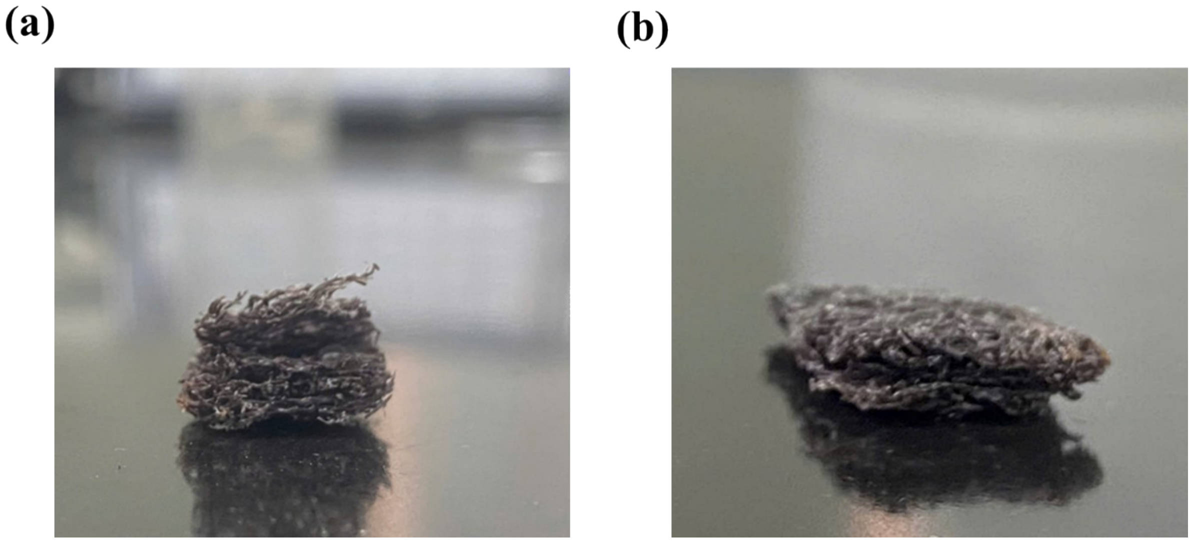

Fig S2. Initial structure of P-CLS (a) and after the recovery process by using mechanical methods (b).

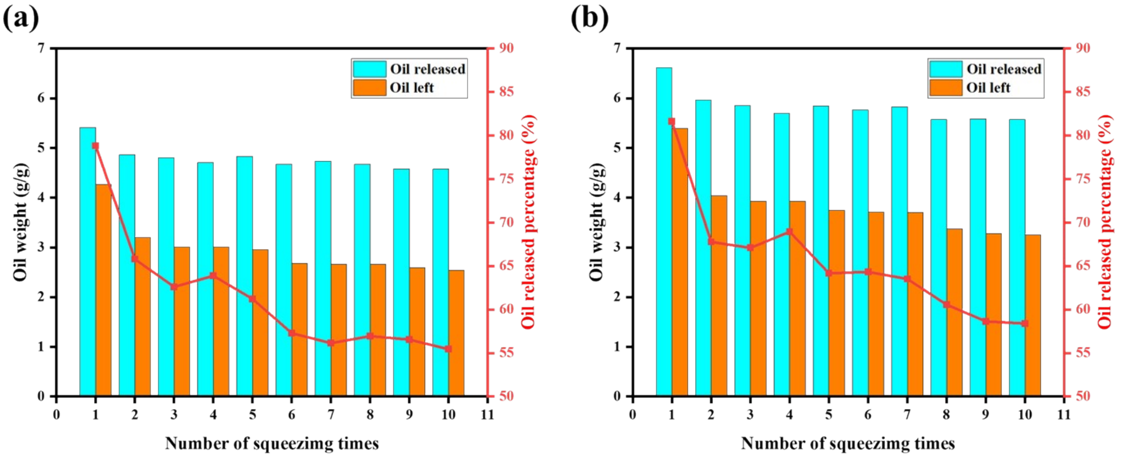

Fig S3. Oil recovery rate from P-CLS in the irradiation (a) and dark condition (b) by using external mechanical methods.

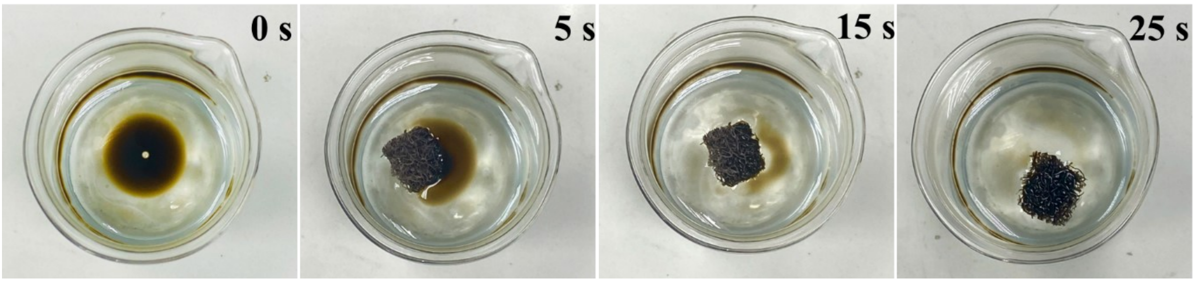

Fig S4. Absorption process of P-CLS
